# Local perceptions and factors determining ecosystem services identification around two forest reserves in Northern Benin

**DOI:** 10.1186/s13002-019-0343-y

**Published:** 2019-12-03

**Authors:** Gerard N. Gouwakinnou, Séverin Biaou, Fifanou G. Vodouhe, Marc S. Tovihessi, Beranger K. Awessou, Honoré S. S. Biaou

**Affiliations:** 1 Laboratory of Ecology, Botany and Plant Biology, Faculty of Agronomy, University of Parakou, 03 BP 125, Parakou, Republic of Benin; 2 Laboratory of Economic and Social Dynamics Analysis, Faculty of Agronomy, University of Parakou, 03 BP 125, Parakou, Republic of Benin; 3 Direction Générale des Eaux, Forêts et Chasses (DGEFC), BP 393, Cotonou, Republic of Benin

**Keywords:** Ecosystem services, Local perceptions, Targeted conservation education, Local community, Benin

## Abstract

**Background:**

Ecosystems provide humanity with goods and services known as ecosystem services. The value of these services represents a basis for political decision-making. To be sure that these decisions are made on a valid basis, policymakers require an understanding of the biophysical processes involved**.** This study was carried out around two forest reserves (Alibori-Supérieur and Ouénou-Bénou) in Northern Benin. It aimed to highlight the knowledge of the surrounding communities and their perceptions about the importance of the ecosystem services provided by these forest reserves as well as the factors that influence their knowledge and perceptions.

**Methods:**

Primary data were collected from 25 group discussions in 25 villages surrounding the forest reserves based on predefined ecosystems services of the Millennium Ecosystems Assessment (MA). Multiple linear regression models were used to examine how socio-economic characteristics of the communities influenced the ecosystem services identification rate. Perceptions of importance, levels of satisfaction, and trends of services provided were analyzed using descriptive statistics.

**Results:**

Our results showed that education level, poverty index, household size, and proximity to forests played an important role in the variation in knowledge of ecosystem services (*P* < 0.05). Provisioning services (such as crops supply, fuelwood, lumber, wild food, and medicinal plants) were mostly identified by the poorest villages located very close to the forests (*P* < 0.05). The importance of the provided services for well-being has been unanimously recognized. The most recognized cultural services were education and knowledge facilitation (84%) and spiritual value (76%). Climate regulation (84%) and pollination (84%) were the best-known regulating services. However, supporting services (soil formation and pest regulation) that are important for improving production systems were unknown to the communities.

**Conclusion:**

Education level, poverty index, and village proximity to the forest were important predictors of regulating and supporting services identification. But use of non-tangible services by local rural communities will require more emphasis on targeted environmental education specifically designed according to the needs of each group.

## Background

In many respects, tropical forests are valuable reserves for local populations [1]. They play a vital and well-known role because of the ecosystem services they provide. These services include timber production [2], non-timber forest products [3], carbon storage [4–7], and many other regulating services. Tropical forests also contribute to biodiversity conservation, climate change mitigation and adaptation [8, 9], resilience [10], water and soil protection, agricultural production [9], and food security [11]. The reliance upon these services (formally known as ecosystem services) makes human beings dependent on the immediate ecosystems for their subsistence [12]. Despite this recognized role of forests in sustaining livelihoods, deforestation is progressing at an alarming rate around the world [13]. Disturbances induced by human activities are resulting in gradual biodiversity loss from forests [14] with the subsequent impact on their structure, ecological functions, and services provision [15]. Biodiversity loss, climate change [16], and pollution are reducing the ability of forests to provide environmental goods and services that support food security and other human needs. The functions and services of ecosystems depend on their state, and also on their utility and the extent of pressure that societies exert on them in a given biogeographic and geo-economic context [17]. Both locally and globally, human societies are modifying ecosystems [18] while deriving economic benefits or adapting to environmental changes [19, 20]. Faced with the widespread ecosystem degradation in the world, the scientific community has reiterated the debate on the relationship between humankind and its environment. In this debate, the notion of ecosystem services is occupying an increasingly important place.

The idea of the services provided by ecosystems to humanity is not new [21]. The concept appeared with authors such as Westman [22], Ehrlich and Mooney [23], and Costanza et al. [24]. Their works pointed out the importance and diversity of services provided to humans by ecosystems, how human activities are degrading the ecosystems, and the impossibility of substituting these services once they are lost. Hence, using the concept of an ecological function or service delivered by ecosystems and the monetary valuation of these services, they intended to alert public opinion and governments about the importance of sustainable ecosystems functioning. This idea attracted the attention of the public and led to the emergence of the concept of ecosystem services. Later on, the Millennium Ecosystem Assessment (MA) completed the endorsement of this concept and propelled its growth outside the scientific sphere [25]. Thereon, the scientific and policy implementation of this concept has given birth to several recent initiatives including the creation of the Intergovernmental Panel on Biodiversity and Ecosystem Services (IPBES), the Economics of Ecosystems and Biodiversity (TEEB), the publication of institutional reports such as the Food and Agriculture Organization (FAO) report [26], and an increasing number of scientific publications devoted to the concept [27, 28]. All these initiatives were aimed at promoting the notion of ecosystem services in a more operational context of biodiversity conservation policies and projects [29]. However, this concept remains elitist and accessible only to experts. It is unclear how local communities, who are the main actors in the forest dynamics and the first direct beneficiaries of ecosystem services, have internalized the concept. The critical importance of taking into account local knowledge and perceptions has been pointed out by several authors as a basic tool in decision-making policy for ecosystem protection, sustainable resource management, and livelihoods [15, 30–40]. The MA [25] in its evaluation report also found that local ecological knowledge was relevant for addressing the issues of unsustainable management of ecosystem services. According to Willock et al. [41], knowledge influences behavioral attitudes and individual intentions. For example, a farmer who has extensive knowledge of the consequences of using insecticides on insect populations (pollinators and natural pest control agents for crops production) [42] will develop behaviors to minimize their adverse effects [41]. Also, the benefits gained from ecosystems by the community are sometimes ignored, wrongly understood, wrongly perceived, or perceived in different ways [35]. Factors affecting people’s dependence on forests or their attitudes towards forest management or conservation have been examined extensively using both spatial and social variables. From a socio-economic viewpoint, a number of studies have revealed that educated people, as well as poor people, valued forest ecosystem services more, albeit sometimes in different ways [34, 43]. Demographic characteristics, family size, gender, and age have also been reported to influence the dependence of local communities on the forest and thus their perceptions of ecosystem services [43, 44]. For example referring to age class, Moutouama et al. [44] reported that adults and old people were more likely to perceive supporting services in Atacora Chain of Mountains in Benin than the young who are less involved in farming activities. From a spatial or geographical location perspective, several studies have shown that a location can change the way a household depends on forest resources [43–46]. Kinzer [47] termed this the “spatial zone of influence.” This is often expressed as a distance in terms of a few kilometers between two rural households [43] to several kilometers between villages and towns [44]. In any case, the influence was shown to decrease with increasing distance from the forest edge, and this decrease was not linear [45]. Most of the time, people close to the forest rely more on forest resources for their livelihood. Consequently, goods provided by the forests (provisioning services) are more important for them. However, distance from forest edge may not be enough to explain the attitude or perception of users of forest resources. The influence of demographics characteristics and/or household level economics also comes into play. This is generally termed the social “zone of influence” [47]. Poor households tend to be most dependent on forest resources and may regard provisioning services as the most important compared to wealthy households. Conversely, educated people value more regulating services [34]. By acknowledging the role that perception plays in shaping local livelihoods and the sustainable management of natural resources, this study aims to understand the local knowledge and perceived importance of ecosystem services provided by Alibori-Supérieur and Ouénou-Bénou forest reserves in Northern Benin and the socio-economic factors that affect the identification of these ecosystem services. We assumed that socio-economic variables, as well as distance from the forest, would be important predictors of the level of awareness of the ecosystem services provided by forests. This study will serve to emphasize the importance of ecosystem services knowledge and perceptions in sustainable forest management for the benefit of forest-dependent communities. From this perspective, the study aims to answer the following research questions: (1) How do communities around the studied forests identify and perceive the importance of ecosystem services? and (2) Which socio-economic factors explain the variations in the identification of ecosystem services? Finally, we discuss the implications of our findings for the sustainable management of forests and the design and implementation of conservation education in order to improve communities’ attitudes towards natural resources management.

## Methods

### Study area

**Fig. 1:**
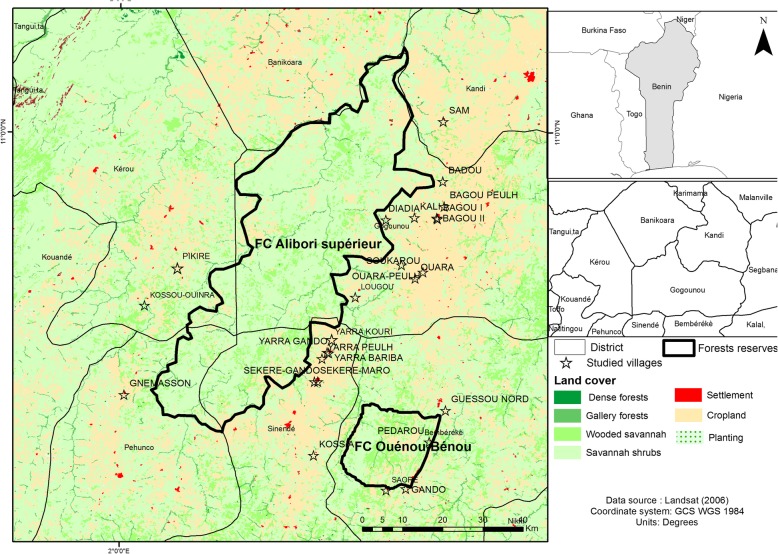
Location of the study zone and sampled villages

The study was carried out in Northern Benin around Alibori-Supérieur and Ouénou-Bénou forest reserves. These forests are located between 1° 55′ and 2° 50′ East longitude and between 10° 05′ and 11° 20′ North latitude. The villages involved in the study were located in the neighboring municipalities and covered 502,356 ha (Fig. 1). This zone mostly belongs to the Sudanian area and is characterized by a uni-modal rainfall regime with a northern gradient ranging from 900 to 1200 mm [48]. Agriculture and livestock were the main activities extensively practiced [49]. Cultivation of cotton, a cash crop, was highly developed and induced a particular agricultural dynamic to the study area [49]. About 97,986 inhabitants lived within a 10-km radius around both forest reserves and were distributed in 11,316 households in 27 villages [50, 51]. The poverty index varied from 21 to 61% [47, 48]. The majority of people were rural with a high dependence on forest ecosystems for their livelihoods. Bariba and related (Gando) (37.3%), Fulani (33.0%), and Dendi (20.1%) were the main socio-cultural groups [47, 48].

### Sampling

The current investigation involved 25 group discussions held in villages around Alibori-Supérieur and Ouénou-Bénou forest reserves from July to August 2017. The number of villages selected for the surveys was based on their relative proximity to the forests (within 10 km from the forest borders). A total of 25 administrative villages including five around Ouénou-Bénou forest reserve and 20 around Alibori-Supérieur were randomly selected. In each participating village, interviews were held with the local community through a group discussion. Each discussion group consisted of 10 to 15 people per village. A total of 285 people participated in the discussions held in the 25 villages. Participants were from different socio-professional groups (farmers, pastoralists, loggers) and socio-cultural groups living in the village. The dominant socio-cultural groups in the surveyed area were Bariba, Gando, and the Fulani.

### Data collection

Twenty-nine (29) ecosystem services grouped into three categories adapted from the four categories of the Millennium Ecosystem Assessments [25] by Zhang [42] were used as a basis for discussion. They were made of ten (10) provisioning services, eleven (11) supporting and regulating services, and eight (08) cultural services. The discussion in each village was held with a local field animator after he had been sensitized to the objectives of the study and ecosystem services concept. The group discussion focused on the knowledge of each of the presented services. Participants in each group were presented with a structured group interview to gather knowledge about the different services provided by the classified forests near to which they resided. To ensure that the number of services identified by each group was recorded accurately, feedback was given with the help of a facilitator. This feedback allowed participants to express their agreement or disagreement about the ecosystem services provided. Once consent was given on the number of services identified, participants were asked to rate the importance of each service (hereafter referred to as a perception) on a scale of 1 to 3 (with 1 as not important and 3 as very important) and the trend of supply of each identified ecosystem service in the last 5 years. Then, the level of education of each participant in the focus group was noted. In this study, a participant was considered as educated when he had attended a secondary school for at least 1 year. The education rate of a considered village was calculated as the number of educated individuals out of the total number of individuals in the discussion group. The distance from each sampled village to the forest edge was determined using the buffer tool of ArcGIS 10.3.1. Additional socio-economic variables such as average household size and poverty index for each village were obtained from the national census [50–52].

### Data processing and analysis

Descriptive statistics were used to characterize the ecosystem services identification level, as well as the importance and trends perceived in the last 5 years. Multiple linear regression models were used to examine how the characteristics of the communities influenced the ecosystem services identification rate. As dependent variables, three indices of ecosystem services identification were used [42]: (i) the number of provisioning services identified out of the total number of provisioning services (*N* = 10); (ii) the number of regulating and supporting services identified out of the total number of regulating and supporting services (*N* = 11), and (iii) the number of cultural services identified out of the total number of cultural services listed for the interview (*N* = 8). As explanatory (independent) variables, we considered the dominant socio-cultural group of the village; the village-forest distance (*D*) using the following typology: close (*D* ≤ 3 km), medium (3 km < *D* ≤ 5 km), and far (*D* > 5 km); the education rate; the average household size; and the poverty index of each village. The Akaike information criterion (AIC) was used to compare the models [53] using the MASS package [54]. For reasons of parsimony, the model with the smallest AIC value and the smallest factor number was selected [55]. This was possible thanks to the ΔAIC calculation for each model as the difference between the AIC of a given model and the minimum AIC (ΔAIC_i_ = AIC_i_ − AIC_min_). The model with ΔAIC < 2 was considered as the best [56]. Statistical analyses were performed using R-3.4.1 [57]**.**

## Results

### Ecosystem services identification rate in surveyed villages

**Fig. 2:**
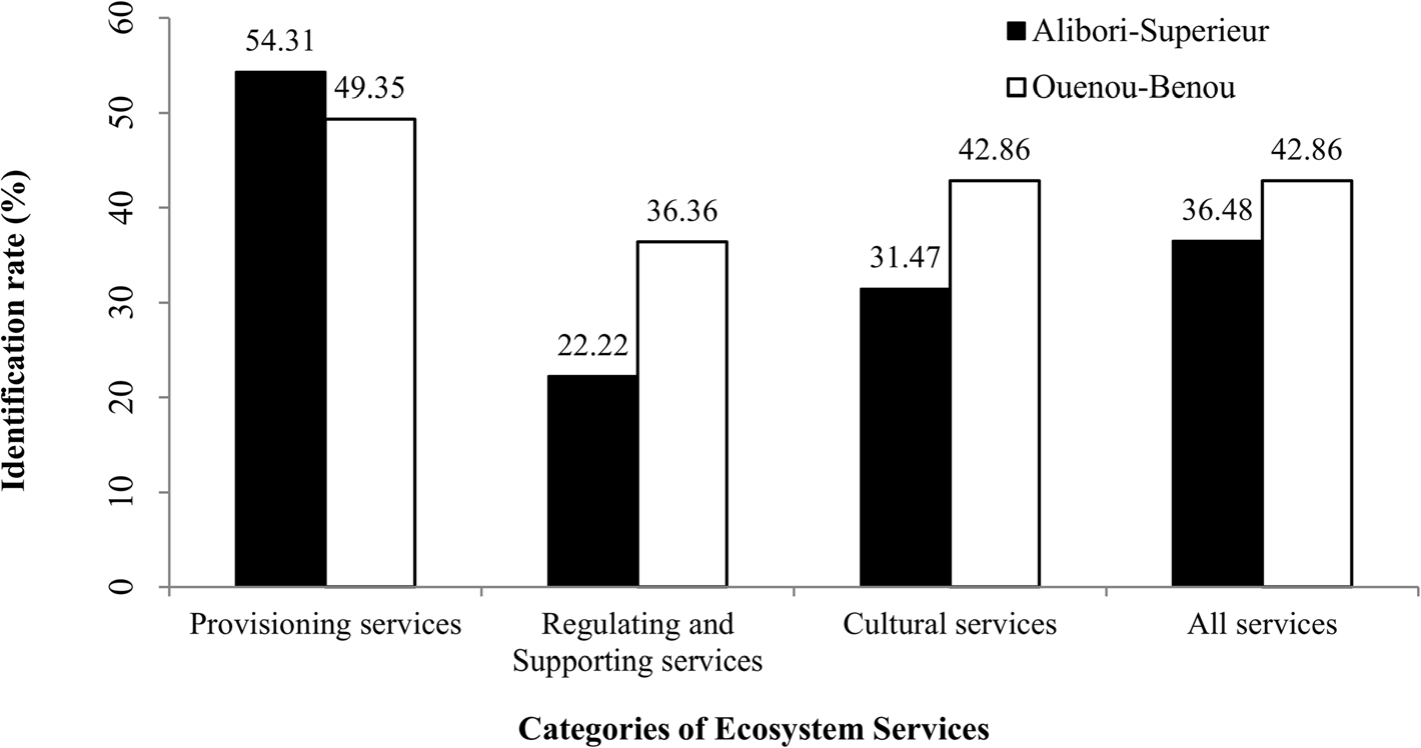
Ecosystem services identification rates grouped by category

**Fig. 3:**
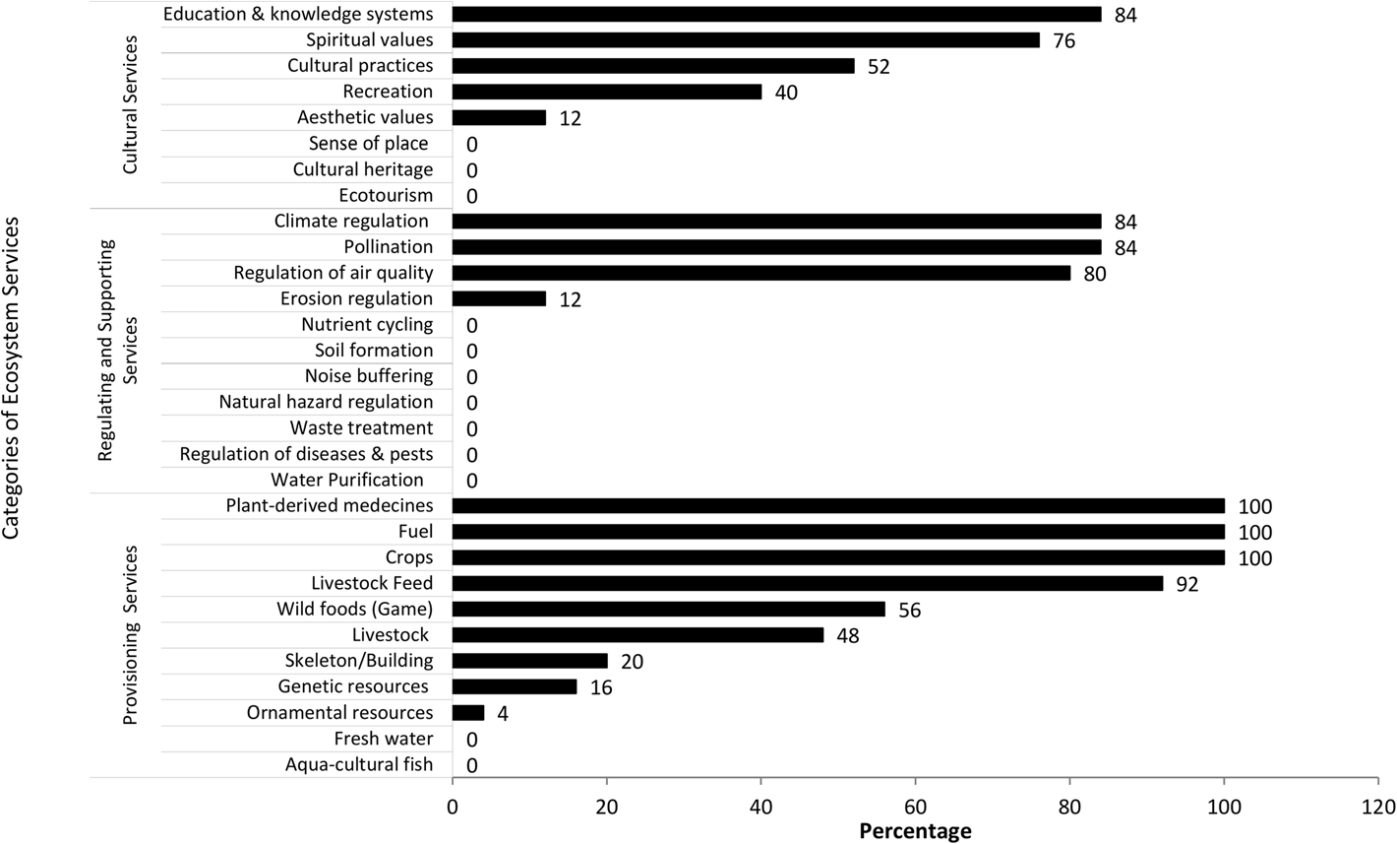
Identification rate for each ecosystem service

In general, provisioning services were highly identified, followed by cultural services and then regulating and support services (Fig. 2). Geographically, it appeared that the local community around the Ouénou-Bénou forest reserve (42.85%) identified more ecosystem services than those around the Alibori-Supérieur forest reserve (36.48%; Fig. 2). Provisioning services such as crop supply, fuelwood, and medicinal plants were identified in all villages around both forest reserves. Food supply, livestock improvement (92%), wild food supply (non-wood forest products) (56%), and lumber (20%) were also well identified in the surveyed villages. Regulating and support services such as pollination (84%), climate regulation (84%), and air regulation (80%) were all identified but at different rates from one village to another (Fig. 3). Erosion control was identified by only 12% of participants in the surveyed villages. More than 50% of the participants in surveyed villages were aware that the forest enabled their cultural practices, contributed to the maintenance of the spiritual value, and constituted a system of knowledge and education (Fig. 3).

### Perceptions of ecosystem services importance and their trends in the last five years

The local communities of both forest reserves perceived provisioning services as the most important (Table 1). For these communities, the supply of crops and fuelwood contributes to their well-being. The perception was similar for services such as animal production improvement (92%), medicinal plants supply (92%), and wild animal food supply (36%) (Table 1). The importance of genetic (16%) and ornamental resources (4%) were weakly emphasized. With regard to regulating and support services, air quality (80%) and pollination (76%) were considered very important (Table 1). Cultural services such as knowledge and education systems and the spiritual value were considered important in 60% of villages surveyed.

**Table 1:** Perceived importance of ecosystem services by villages and trends in the last 5 years

Perception	Importance of ES			Trends of ES			
	Very important	Not important	Do not know	Improving	Declining	No change	Do not know
Provisioning services							
Livestock feed	36.00	8.00	4.00	8.00	24.00	8.00	8.00
Livestock	92.00	0.00	0.00	40.00	36.00	12.00	4.00
Building/frame	20.00	0.00	0.00	0.00	20.00	0.00	0.00
Crops	100.00	0.00	0.00	32.00	36.00	28.00	4.00
Fuel	100.00	0.00	0.00	12.00	84.00	4.00	0.00
Wild foods (plants, animals)	48.00	8.00	0.00	4.00	48.00	0.00	4.00
Genetic resources	16.00	0.00	0.00	4.00	8.00	4.00	0.00
Ornamental resources	4.00	0.00	0.00	4.00	12.00	4.00	0.00
Plant-derived medicines	96.00	0.00	4.00	12.00	68.00	20.00	0.00
Regulating and support services							
Pollination	76.00	0.00	8.00	12.00	44.00	4.00	24.00
Regulation of air quality	80.00	0.00	0.00	8.00	20.00	32.00	20.00
Erosion regulation	12.00	4.00	0.00	0.00	12.00	0.00	4.00
Climate regulation	64.00	0.00	20.00	4.00	44.00	12.00	24.00
Cultural services							
Education and knowledge systems	80.00	0.00	4.00	0.00	64.00	8.00	4.00
Asthetic value	4.00	0.00	8.00	0.00	4.00	0.00	8.00
Spiritual value	64.00	0.00	12.00	0.00	40.00	20.00	16.00
Cultural practices	36.00	0.00	16.00	0.00	24.00	8.00	20.00
Recreation	36.00	0.00	4.00	0.00	20.00	8.00	12.00

*Legend*: *ES* ecosystem services

The majority of local communities reported declining trends in the last 5 years especially for services such as fuelwood, medicinal plants, and wild food (game) supply (Table 1). Despite the low rates of identification of regulating and support services, 44% of villages reported a decline in services related to climate regulation and pollination. Air regulation (20%) was reported to have declined by participants in the studied villages. This was perceived through frequent heatwaves. Although the forest was reported to offer very few cultural services, 64% of villages reported a declining trend in services related to the education and knowledge system. The decline in spiritual value was noted by only 40% of villages (Table 1).

### Socio-environmental factors affecting ecosystem services identification rate

#### Provisioning services

**Fig. 4:**
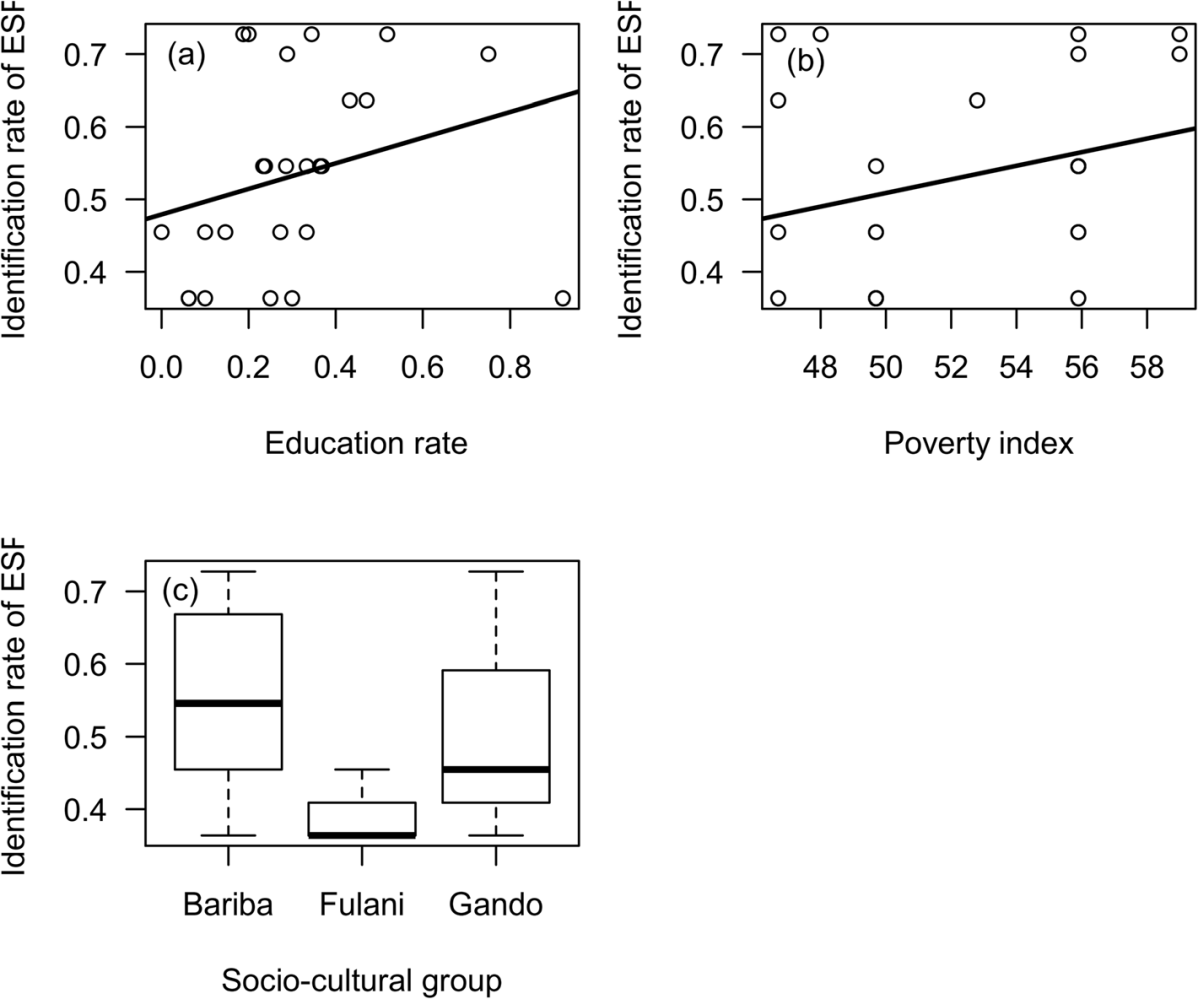
Identification rate of provisioning services according to socio-environmental factors. Legend: **a** education rate; **b** poverty index; **c** socio-cultural group; *ESP* provisioning ecosystem services

Participants of the poorest villages identified significantly more provisioning services (*P* < 0.05; Table 2). Only the Fulani socio-cultural group identified significantly fewer provisioning services compared to other socio-cultural groups (Fig. 4c). Moreover, the distance from the village to the forest edge had a significant effect on the identification rate of services (*P* < 0.05; Table 2). The closer and moderately closer communities to the forest (located within 5 km of the forest’s edge) identified significantly more provisioning services than those located at more than 5 km (*P* < 0.05; Table 2). The education rate did not significantly impact the rate of provisioning service identification (*P* > 0.05; Table 2; Fig. 4b).

**Table 2:** Effects of socio-economic factors on the identification rate of ecosystem services

	Ecosystem services								
	Provisioning services			Regulating and supporting services			Cultural services		
Variables	Estimate	*t* value	Pr (> |t|)	Estimate	*t* value	Pr (> |t|)	Estimate	*t* value	Pr (> |t|)
Constant	− 2.59845	− 2.245	0.03837*	− 1.21308	− 0.946	0.35915	− 2.52647	− 0.939	0.3635
Moderately close	3.469771	2.41	0.02756*	2.87	2.118	0.05132.	1.78966	0.559	0.585
Very close	3.994596	3.205	0.00519**	4.03242	3.491	0.00329**	4.31767	1.556	0.1419
Education rate	0.062971	0.796	0.43714	− 4.57245	− 1.934	0.07216.	− 3.24144	− 1.386	0.1876
Poverty index	0.048881	2.289	0.03517*	0.0295	1.307	0.2109	0.05773	1.213	0.2451
Household size	0.002533	0.135	0.89449	− 0.0349	− 1.447	0.16858	− 0.0836	− 1.209	0.2468
Moderately close: poverty index	− 0.060507	− 2.341	0.0317*	− 0.04975	− 2.045	0.05886.	− 0.02079	− 0.358	0.7256
Very close: poverty index	− 0.071471	− 3.232	0.0049**	− 0.07214	− 3.51	0.00316**	− 0.07151	− 1.448	0.1698
Moderately close: education rate							− 1.94736	− 2.491	0.0259*
Very close: education rate							− 1.31318	− 2.176	0.0472*
Education rate: household size							0.55292	1.874	0.0819.
Education rate: poverty index				0.05256	1.607	0.12892			
Education rate: household size				0.21173	2.238	0.04084*			

*N* = 25 villages

* *P*-value < 0.05; ** *P*-value < 0.01

#### Regulating and supporting services

**Fig. 5:**
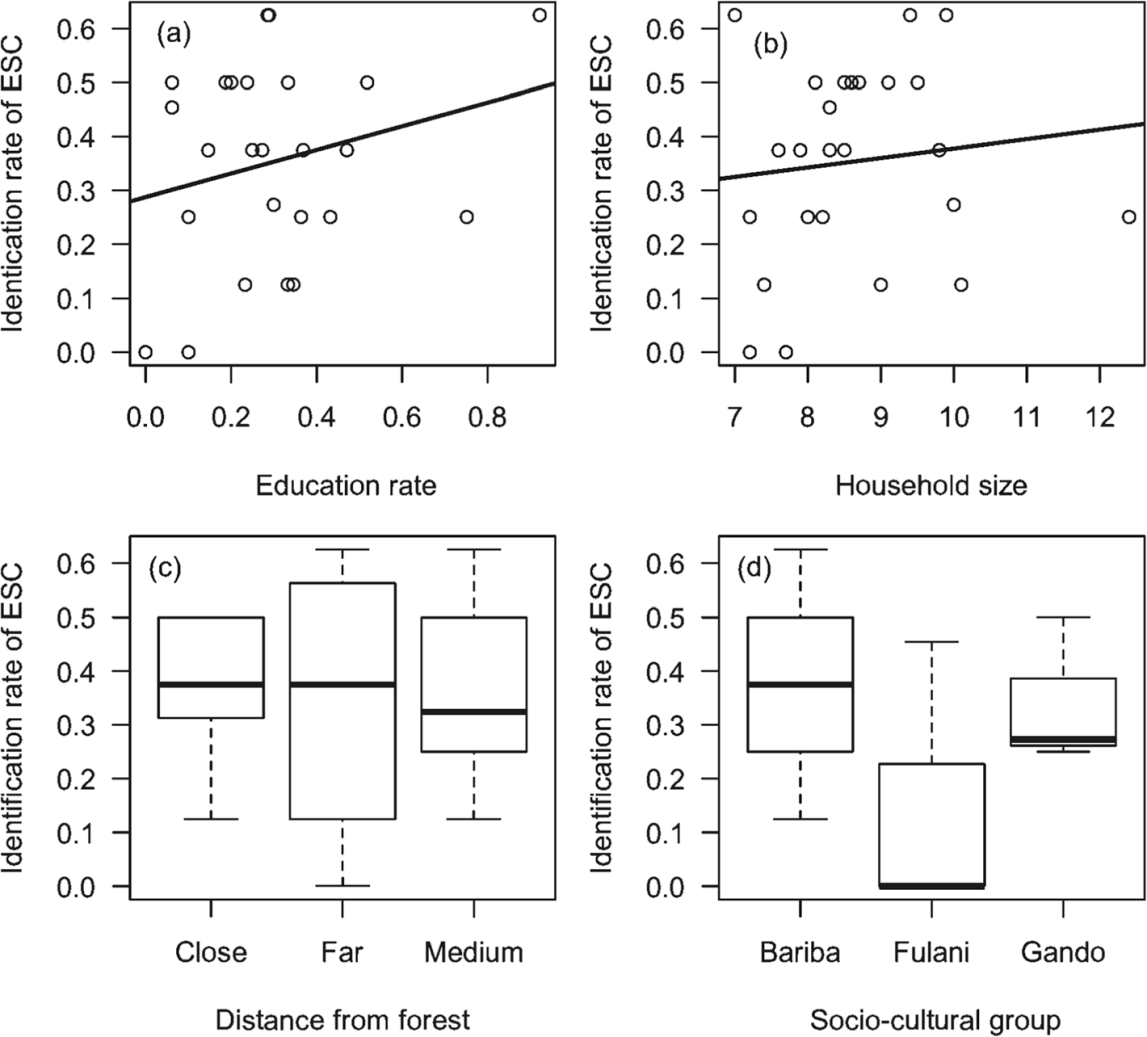
Rate of identification of regulating and support services according to socio-environmental factors. Legend: **a** education rate; **b** poverty index; **c** distance from forest; **d** socio-cultural groups; *ERS* regulating and support ecosystem services

The results revealed that the poorest communities (high poverty index) identified significantly fewer support and regulating services (*P* < 0.05; Table 2; Fig. 5c). Here again, the Fulani socio-cultural group identified these services poorly compared to other socio-cultural groups (Fig. 5b). It also appeared that villages within 5 km of the forest were better at identifying the regulating and support services than those living further away (Fig. 5a). In poor villages close to the forest, regulating and support services were less identified. Education rates had a positive impact on the identification of regulating and support services (*P* < 0.05; Table 2; Fig. 5d). The higher the education rate, the higher the identification rate.

#### Cultural services

**Fig. 6:**
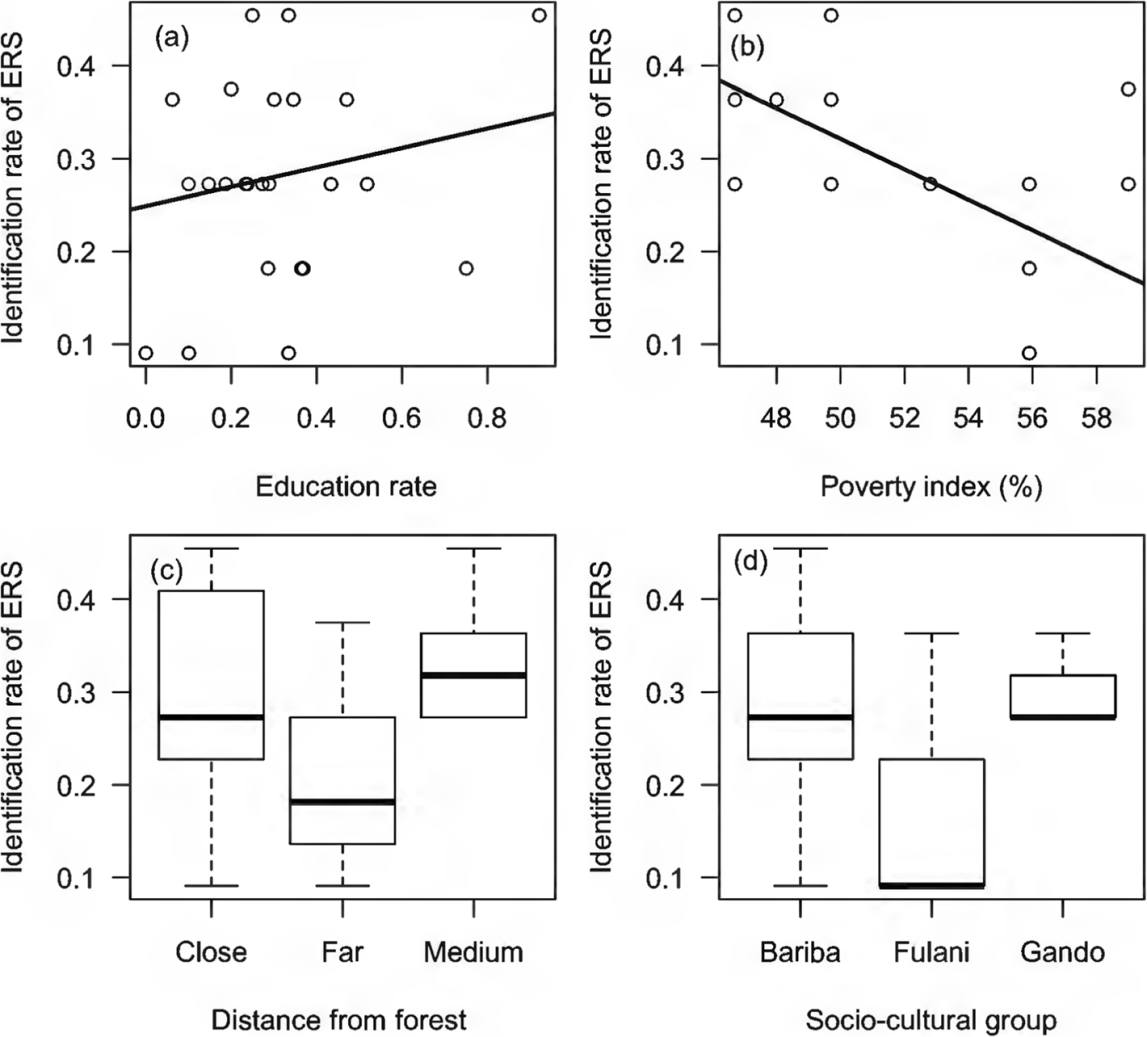
Rate of identification of cultural services according to socio-environmental factors. Legend: **a** education rate; **b** household size; **c** socio-cultural groups; **d** distance from forest; *ESC* cultural ecosystem services

The results showed that cultural and religious services rates varied by village (*P* > 0.05; Table 2). The higher the education rate in a village, the higher the cultural services identification rate (Fig. 6a). Also, the interaction between the education rate and household size had a significantly positive effect on the identification rate (*P* < 0.05; Table 2). However, in moderately close villages, communities with a high education rate did not significantly identify the cultural services (*P* < 0.05; Table 2), suggesting that distance to the forest edge was a determining factor in the cultural services identification. Fulani communities showed little interest in the cultural services offered by these forest ecosystems compared to the other two socio-cultural groups (Fig. 6a).

## Discussion

In this study, we investigated the knowledge and local perceptions of ecosystem services by people living in villages surrounding two forest reserves. We also analyzed the socio-cultural and geographic factors determining their knowledge and perceptions. We finally discussed the implications of our findings for the design of conservation education based on ecosystem services for the sustainable management of forests and natural resources.

### Factors determining ecosystem services identification

Prior studies have documented the importance of local ecological knowledge and how the perceptions of the immediate environment by the local population influence their decision to conserve natural resources. For example, Gaoué and Ticktin [58] reported how the traditional Fulani practice of *sopoodu* can provide a basis for sustainable management of *Khaya senegalensis* (Desr.) A. Juss. plantations. In contrast, Gouwakinnou et al. [59] reported a folk perception about how some farmers use the appearance of the back of a tree to determine its sex. Unfortunately, this turned out to be a false clue likely to result in the poor management of *Sclerocarya birrea* (A. Rich.) Hochst. populations. From these studies, it appears that the attitudes of local users about whether or not to conserve a resource are determined by the local ecological knowledge of a resource, its perception, and its usefulness. In this study, we found that the concept of ecosystem services, which is clearly formalized in the scientific world, has not been totally mastered at the level of local communities. The provisioning services were the most identified services by people from villages closest to the forest reserves. This could be an indicator of the dependence of these communities on these services for their livelihoods. These results corroborate those of Muhamad et al. [46] and Sodhi et al. [34], who found that people living near to forests tended to have a good appreciation of ecosystem services in West Java and Indonesia, respectively.

It appeared that the identification rate of provisioning services was higher compared to other services. Similar observations were made by previous studies [42, 46, 60], which can be explained by the tangible nature of provisioning services. Firewood was a necessity for the local community as a source of primary energy from forest ecosystems and contributed to economic income through firewood markets. A variety of foods such as fruits and other woody plant organs from the forest also contributed to food security in the study area. The community drew micronutrients from wild foods. Moreover, plant parts were used in traditional medicine. Therefore, the high level of provisioning service identification was justified by their direct implications on the physical, economic, and social well-being of the community [43]. Moreover, the level of poverty was an important factor in identifying provisioning services, confirming that the poorest people relied more on provisioning services from ecosystems for their daily subsistence.

Also, although regulating and support services are of paramount importance for ecosystem health and improved agricultural production, our investigation showed that they were identified less frequently by the local community. This lack of knowledge of these services can be explained by the low level of education of local communities.

Education rate and population income levels are important variables determining the identification of regulating and support services. This suggests a close link between forest conservation and the economic status of services provided by ecosystems. The poorest and least-educated local populations were unaware, for example, of the close links between agriculture and the services provided by forests. In fact, according to Ryan et al. [61], nutrient cycling regulating and soil erosion are often identified, although pest control may be important in some cases. Indeed, these services are linked to non-visible ecological processes [62], and their mastery is intimately linked to a specialized education. Martin-Lopez et al. [63] and Poppenborg et al. [64] found that education and economic levels have a positive influence on knowledge and attitudes towards regulating and support services. Nevertheless, in most surveyed villages, pollination and climate regulation were frequently identified by local people as services provided by forests. This finding is opposed to the results of Zhang et al. [42] from the climatic zones of Nigeria. The identification of these regulating services in our study can be explained by the various initiatives of sensitization and training in beekeeping in the study area by conservation-based agencies or NGOs. These initiatives often raise the awareness of local communities about the importance of bees in improving the production of cultivated crops. This reflects the positive impact of awareness-raising policies in changing attitudes and suggests the influence of knowledge on the attitudes and behavioral intentions of an individual [41]. Although the pollination service was identified, the importance of insects in pest control in general seemed not to be well known by the local communities. Therefore, these pollinator services should be maintained by good conservation and the management of sufficient resources for wild pollination in agricultural landscapes [65]. Clean water supply through the forest ecosystem was not identified. This revealed the lack of awareness about the important role played by trees and forests in the regulation of water flow and the provision of clean water at a watershed level.

The cultural services identification rate by communities around the two forest reserves was low in comparison with other categories of services. Although forests and trees are known to have many spiritual values in Africa [61], access to forest reserves is generally restricted, which prevents some cultural practices from taking place within them, unlike in sacred groves. Moreover, unlike wildlife reserves, forest reserves are not intended to attract tourism or educational activities, which could enhance their cultural services value.

Thus, traditional ecological knowledge about the functioning of forest ecosystems could be a means of conserving the integrity of the forest ecosystems on which the endogenous community depends for its well-being [39]. The success of such knowledge could be the fruit of new management strategies that depend on the contribution of actors in charge of forest conservation.

### Local perceptions of ecosystem services importance and implications for conservation education design and forest conservation policy

Social preferences for the provisioning services vary from one society to another [66]. But the most important services can be identified by taking into account the opinions of stakeholders from several social groups [67]. These preferences can then be used for the planning of local development [68]. Provisioning services were considered the most important by the studied villages, which demonstrated the dependence of households on provisioning services [69]. The importance of forests as a genetic resource provider was not identified, suggesting a lack of community involvement in the conservation of genetic material of some species. Similar observations were made by Zhang et al. [42] in the climatic zones of Nigeria. In addition, services such as soil formation and nutrient cycling, which are a fundamental support for agricultural production, were not very important to the studied villages despite the predominance of farmers. Only air quality and pollination were perceived as important.

The decline in provisioning services, as reported by studied villages, was attributable to poor land use that did not permit sustainable management of resources. Such comments from local communities pointed to the degraded state of the two forests reserves. It is important to implement an awareness policy on good practices for the sustainable management of subsistence resources for local communities. Also, cultural services such as the knowledge and education system and spiritual value were considered important by the local community. The perceived importance of these cultural services should be reflected in the protection of these ecosystems. According to Ahammad et al. [70], the cultural importance of forests reflects the positive attitudes of local populations for its conservation. The perception of these local communities about the value of ecosystem services should be taken into account when making decisions about the management of these ecosystems. Without understanding the societal values and integrating them into decision-making, it will be difficult to improve synergies and minimize trade-offs between services in the studied ecosystems. Given the diversity of ecosystem services valued by the local communities, there are opportunities to conserve plots in these forests in order to optimize the provision of provisioning, regulating, and, above all, cultural services to improve educational value.

## Conclusion

This study was interested in the perceptions and factors determining the identification of ecosystem services. Of 29 listed services, 18 were identified by at least one village. The provisioning services essential for the livelihoods of the local communities were most identified, followed by the cultural services. The regulating and support services were the least identified. Factors such as educational level were crucial in the identification of regulating and support services. Household size and distance from the forest were determinants of the ability to identify provisioning services. The importance and decline of provisioning services were identified more frequently by communities than regulating and support services and cultural services, which appeared to be more abstract. Because the sustainable maintenance of provisioning services depends on the healthy functioning of ecosystems and the good provision of regulating and support services, advocacy should be made to raise the awareness of the local communities on the importance of regulating services. Because regulating services are not tangible, sensitization campaigns and the integration of the notions of ecosystem services in the basic education system would be advisable.

## Data Availability

The datasets used and/or analyzed during the current study are available from the corresponding author on reasonable request.
